# Features of Cross-Correlation Analysis in a Data-Driven Approach for Structural Damage Assessment

**DOI:** 10.3390/s18051571

**Published:** 2018-05-15

**Authors:** Jhonatan Camacho Navarro, Magda Ruiz, Rodolfo Villamizar, Luis Mujica, Jabid Quiroga

**Affiliations:** 1 Department of Mathematics, Escola d’Enginyeria de Barcelona Est. (EEBE), Universitat Politécnica de Catalunya (UPC) Barcelonatech, Campus Diagonal Besòs, Edifici A, C. Eduard Maristany, 10-14, 08019 Barcelona, Spain; magda.ruiz@upc.edu (M.R.); luis.eduardo.mujica@upc.edu (L.M.); 2 CEMOS Research Group, Electrical, Electronics and Telecommunications Engineering School, Universidad Industrial de Santander (UIS), Bucaramanga 680002, Colombia; rovillam@uis.edu.co (R.V.); jabib@uis.edu.co (J.Q.)

**Keywords:** piezodiagnostics, Baseline Models, Damage Statistical Analysis, principal component analysis, structural damage detection

## Abstract

This work discusses the advantage of using cross-correlation analysis in a data-driven approach based on principal component analysis (PCA) and piezodiagnostics to obtain successful diagnosis of events in structural health monitoring (SHM). In this sense, the identification of noisy data and outliers, as well as the management of data cleansing stages can be facilitated through the implementation of a preprocessing stage based on cross-correlation functions. Additionally, this work evidences an improvement in damage detection when the cross-correlation is included as part of the whole damage assessment approach. The proposed methodology is validated by processing data measurements from piezoelectric devices (PZT), which are used in a piezodiagnostics approach based on PCA and baseline modeling. Thus, the influence of cross-correlation analysis used in the preprocessing stage is evaluated for damage detection by means of statistical plots and self-organizing maps. Three laboratory specimens were used as test structures in order to demonstrate the validity of the methodology: (i) a carbon steel pipe section with leak and mass damage types, (ii) an aircraft wing specimen, and (iii) a blade of a commercial aircraft turbine, where damages are specified as mass-added. As the main concluding remark, the suitability of cross-correlation features combined with a PCA-based piezodiagnostic approach in order to achieve a more robust damage assessment algorithm is verified for SHM tasks.

## 1. Introduction

“Structural health monitoring (SHM) is the integration of a sensory system, a data acquisition system, a data processing and archiving system, a communication system, a damage detection system, and a modeling system to acquire knowledge about the integrity and load worthiness of in-service structures on either a temporary or continuous basis” [1]. Likewise, according to Farrar and Worden [2], SHM can be defined as the process of implementing a damage identification strategy for aerospace, civil, and mechanical engineering infrastructure. The benefits of implementing damage identification strategy through an SHM system are an avoidance of premature breakdowns, a reduction of maintenance costs, continuous remote diagnosis, and economic benefits in terms of an operational life extension. For instance, leakage detection systems, which can be designated through an SHM paradigm, are of great importance in the oil industry, where pipes are critical for oil and gas transportation [3]. Thus, by implementing an SHM scheme, it is possible to mitigate economical losses and environmental damages. Several approaches for pipeline damage detection are detailed in the review presented by Murvay et al. [4]. In [5,6], the applicability of technologies based on acoustic emissions as part of an SHM system is demonstrated. Additionally, fiber optics techniques [7] and statistical processing of pressure measurements [8] have been reported to be effective in SHM systems. Some novelty methodologies for pipe leak damage detection includes piezoelectric measure processing, as is shown in work developed by Feng et al. [9], where a crack detection and leakage monitoring system on reinforced concrete pipe is evaluated. Another example is detailed in [10], where a method for gas pipeline leakage detection based on PZT sensors is proposed.

**Figure 1:**
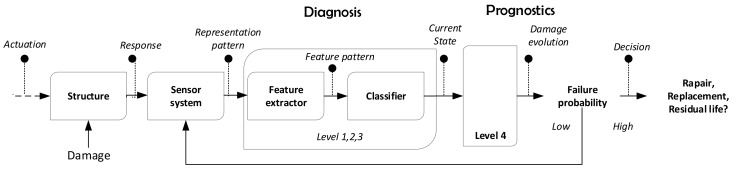
Components of a structural health monitoring (SHM) process for damage diagnosis according to Ooijevaar [11].

As is illustrated above, many SHM approaches have been reported in the literature, where a trade-off between efficiency and accuracy in the diagnosis is one of the main objectives. According to Ooijevaar [11], structural damage diagnosis algorithms include elements summarized in Figure 1.

In general terms, the sensor system obtains the signal signature describing the current state of the monitored structure. Data collected by the sensor network is then characterized through features in order to get a sensible representation to different conditions. These features are exploited by classification, regression, or clustering algorithms with the ability to identify abnormal conditions (i.e., possible damage). Thus, by implementing the scheme depicted in Figure 1, basic SHM levels can be achieved: diagnosis constituted by damage detection (Level 1), location (Level 2), quantification (Level 3), and prognostics by estimating the damage evolution (Level 4), where feature extraction receives most of the attention in the literature [2]. Since the SHM process requires features with high sensitivity to distinguish between undamaged and damaged conditions, this process should be robust to noisy measurements. Thus, feature extraction can be complemented using data cleansing and preprocessing techniques in order to improve diagnosis response of the whole system and consequently to minimize effects due to variable operational and environmental conditions as well as sensor drifts.

On the other hand, there have been numerous recent studies for damage detection within a statistical framework and specially focused on data-driven models. For instance, in order to accurately simulate the complex dynamics of operational wind turbines a bi-component analysis tool is applied on long-term experimental data [12]. Additionally, the authors of [13] propose the use of statistical and modal damage detection methods for the damage detection problem in a small-scale wind turbine. Other authors, such as those of [14], have presented the use of a time-series as a modeling approach to provide an effective and compact global representation of the vibrational response of a structure under a wide span of environmental and operational conditions. As a result, the state of the art indicates that statistically based methods using data-driven approaches have been reported to be successful for structural condition assessment.

In the same way, algorithms based on principal component analysis (PCA) have been reported as a promising approach to detect and locate damages in structures as pipes, civil structures, and aircraft sections, among others. Recently, there have been many instances using PCA for damage detection combined with other physics-based and data-based methods. For instance, a framework for parameter estimation through proper orthogonal decomposition is presented in work developed by [15] validated with simulated experiments on eight-story shear type building. Similarly, in [16], a reduced-order model of a structure based on proper orthogonal decomposition and Kalman filtering is proposed for the online health monitoring of damaged structures. Another example is proposed in [17], where order-reduction of a numerical model is used to track the structural dynamics for thin flexible plates, which are obtained by means of POD and Kalman filters. Thus, according to the existing literature, PCA data-driven models have been suitable monitoring schemes for damage assessment.

In addition to the above damage detection schemes, some proposals have investigated the application of correlation or covariance functions and PCA or SVD methods as a damage detection algorithm with ability to remove data noisiness involved in the SHM process. For instance, the authors of [18] discuss subspace-based methods to manage issues related to intrinsic uncertainty due to finite data length, colored noise, non-stationary excitations, model order reduction, and operational influences evaluated on a prestressed concrete road bridge. Another example is detailed in [19], where detection and localization in a concrete bridge is developed by the application of stochastic subspace-based structural identification. Additionally, the authors of [20] present a damage detection method based on subspace identification concepts and statistical process techniques that are sensitive to small-sized structural damages and suitable for online monitoring. Similarly, the authors of [21] describes mathematical tools to address inverse problems in structural dynamics to develop data-driven approaches for damage detection.

The above literature review motivates the exploration of data-driven approaches, cross-correlation functions, PCA processing, and piezodiagnostic approach in order to improve the overall performance of structural damage detection algorithms. Thus, this paper discusses the advantages of including a preprocessing stage based on a cross-correlation technique as a useful tool for common noise suppression, outlier filtering, and grouping damage types in a PCA-based piezodiagnostic framework. This analysis is included in the developed methodology, which is tested in three experimental setups, where each one has a PZT as an actuator and the others have PZTs as sensors. These data are preprocessed through cross-correlation, and PCA is then implemented for damage detection in order to discriminate the different damage levels. Next, a clustering learning tool is used to validate the robustness of the proposed methodology.

The paper is organized as follow: Section 2 describes the methods used for damage identification, where some details about instrumentation requirements as well as data organization and damage indexes useful for damage monitoring are presented. Section 3 describes the experimental test structures, while Section 4 presents and discusses the results of applying the whole methodology. Finally, Section 5 concludes the main contributions of this work.

## 2. Damage Assessment Elements

The methodology for damage detection used in the present work is based on three main elements: experimental setup, statistical processing, and clustering analysis. Because one of the main objectives of this work is the application of cross-correlation to improve SHM diagnosis, the procedure for its implementation is detailed in the next section. Thus, the necessary tools for condition monitoring are presented.

### 2.1. Hardware and Experimental Setup

**Figure 2:**
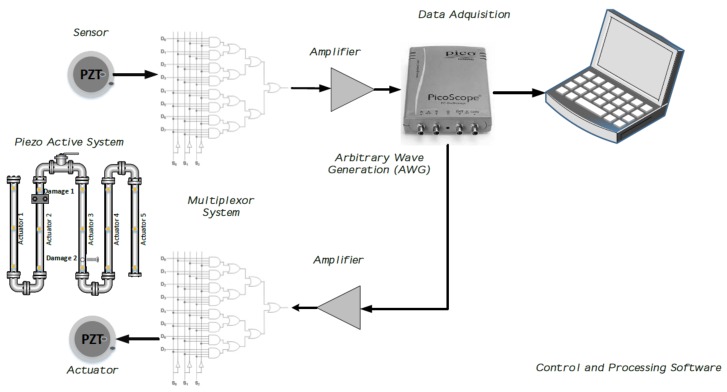
The piezoelectric instrumentation system.

Components of the whole piezoelectric system, whose implementation requires signal conditioning and an acquisition system (i.e., amplifiers, signal generator, multiplexer devices, and software integration, among others) as well as elements for mechanical coupling (i.e., materials to facilitate PZT attachment to the surface structure) are presented in Figure 2. In this research, PZTs are attached to the testing specimens through an adhesive layer of cyanoacrylate. All components of the instrumentation system are managed by means of programming software that controls the command flow. The proposed methodology is experimentally validated in three structures: a carbon steel pipe section, an aircraft wing specimen, and a blade of a commercial aircraft turbine. They are equipped with piezoelectric devices in order to induce guided waves along the surface structure. The carbon steel pipe section facilitates simulating leak and mass-added damage types, while in the other two specimen non-reversible mass-added damage types were recreated. One PZT is excited with a periodic high frequency burst type signal inducing a guide wave and the remaining piezo-devices measure the guided wave response at different locations of the structure. A Picoscope${}^{\mathrm{TM}}$ series 2000 and a 16-Channel multiplexer board comprises the acquisition hardware used to acquire the signals. Arbitrary wave generation (AWG) such as burst type signals is effectuated by means of PicoScope${}^{\mathrm{TM}}$. The system design considers exciting dominant wave modes by operating the actuator PZT element at resonance frequency, which is intended to minimize dispersive behavior and with the purpose of maximum amplitude performance. Guided waves in this study are generated by thin disks of ceramic material (titanium lead zirconate) configured in radial mode. A five-cycle burst-modulated pulse is used to excite the PZT actuator around its resonance frequency ($f_{r}$ 102 kHz). In consequence, many wave-packets corresponding to longitudinal and flexural modes are generated, which represents a highly dispersive pattern as a result of the superposition of several guided waves.

#### 2.1.1. Pipe Section

This test structure is a carbon-steel pipe section with material properties similar to those used in the local industry. Its dimensions are 1 m in length, 2.54 cm in diameter, and 3 mm in thickness with 4″ bridles welded at the ends. On one of the ends, a blind bridle is connected while on the other end, an air source is coupled. The pipe section is equipped with piezoelectric devices distributed along the structure to capture guided wave response. The actuator PZT transducer is located on one of the pipe ends in order to demonstrate the ability of PCA statistical processing to manage the high dispersive performance caused by guided wave bounces due to the elements near the bridles. Additionally, the PCA-based piezodiagnostics approach described in this study is independent from the PZT actuator location. In this pipe section, two types of damages can be studied: leaks and added mass.

**Figure 3:**
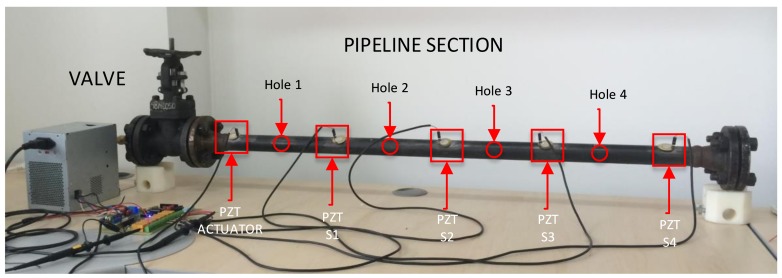
Leak damage type configuration.

The pipe section is depicted in Figure 3. Leaks are induced through elements denominated as $Hole_{i}$. Four quarter-inch holes are drilled along the pipe section wall by means of adjustable screws to control where the leak is produced. A valve is used to set at 80 psi the air pressure from a compressor, which recreates pre-stressed operational conditions and generates flow disturbances in the piezoelectric system. Bolts and other elements used to recreate leak damages are included in the nominal state of the structure and consequently in the statistical baseline model.

**Figure 4:**
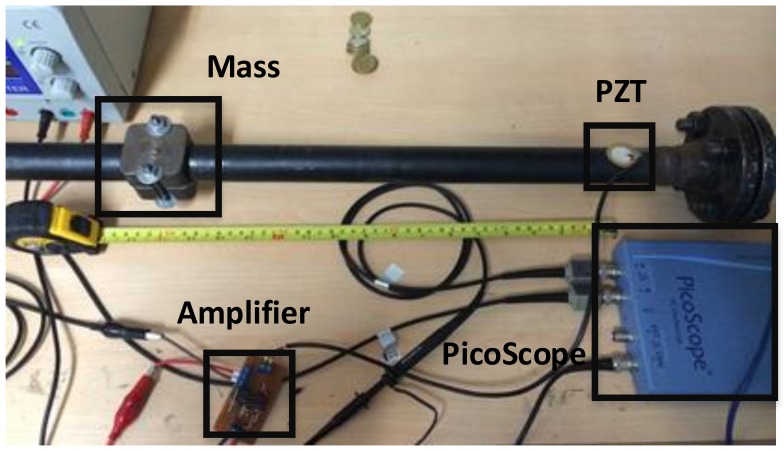
Mass-added experiment mock-up.

In addition to leak conditions, experimental data from mass-added scenarios were used to validate the effectiveness of the methodology. Figure 4 shows the configuration of this type of damage.

According to Figure 4, a special shaped accessory is added to the surface of the pipe section to recreate mass-added damage. In this sense, damage cases are the mass accessory attached to the structure at different locations. The mass occupies 5 cm of the pipe length, which is considered as a source of uncertainty involved in the scenarios configuration.

#### 2.1.2. Aircraft Wing Structure

**Figure 5:**
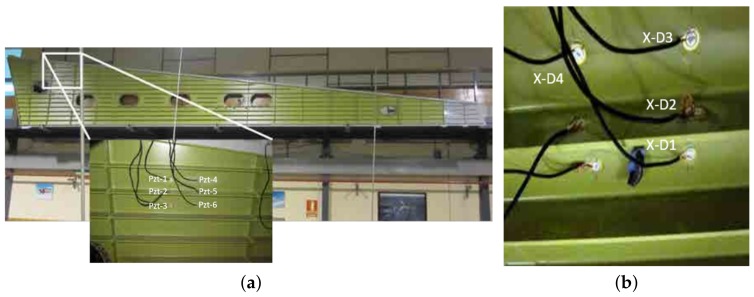
Aircraft wing test structure. (**a**) Skin panel. (**b**) Mass-added damage.

An aircraft wing specimen hosted in the Universidad Politécnica de Madrid (UPM—Spain) was also used to validate the proposed damage assessment methodology. This structure is an aircraft wing panel, which is divided by stringers and ribs as is illustrated in Figure 5a. Two sections of it were equipped with 6 PZTs (two at the upper section, two in the lower section, and two at the rib). Four reversible mass-added damage types were induced in the structure by adding a clay element at different positions according to Figure 5b, where the x-tick symbolizes the damage location and Di is the damage tag.

#### 2.1.3. A Blade of a Commercial Aircraft Turbine

**Figure 6:**
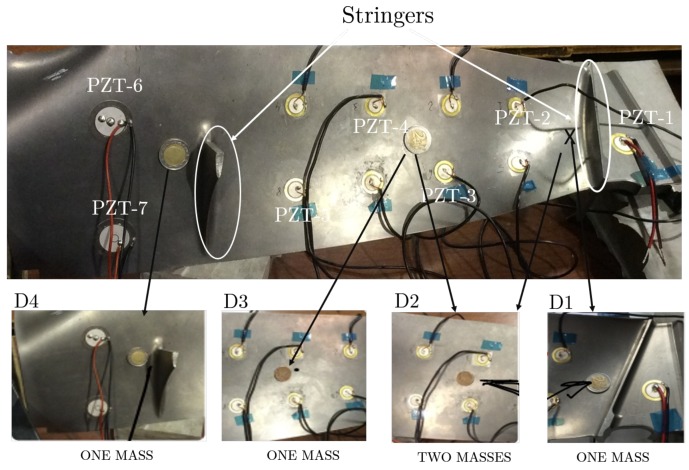
Mass-added damage in the turbine blade structure.

The third specimen used to validate the proposed methodology is a blade of a commercial aircraft turbine, which has an irregular form and includes stringers in both faces (Figure 6). Ten PZTs were attached to its surface, but only 7 of them, located at intermediate positions between the stringers and labeled in Figure 6 as PZT1, PZT2, …, PZT6, were used. The remaining PZT devices are assumed to be part of the structure and taken into account at the baseline model. Four mass-added damage types were simulated in the turbine blade by attaching coins of different denomination and labeled in Figure 6 as D1, …, D4.

According to Figure 6, the damage configuration considers scenarios of different positions, severities, and potential barriers for guided waves. For example, D2 is the addition of two masses at different positions of the surface structure.

### 2.2. Statistical Processing

The second element, the statistical processing of piezoelectric measurements, is developed by preprocessing through cross-correlation analysis and principal component analysis (PCA). The cross-correlation is used as a preliminary cleansing procedure, which is included as a data preprocessing stage in order to minimize the presence of outliers and consequently to improve the discrimination between different types of damage. The statistical processing also includes removing noise and low frequency disturbances from piezoelectric measurements by means of linear detrending analysis. Thus, after statistical processing, PCA is implemented for data fusion, data normalization, and baseline statistic model building, which facilitate the identification of abnormal structural conditions.

#### 2.2.1. Preprocessing through Cross-Correlation Analysis

**Figure 7:**
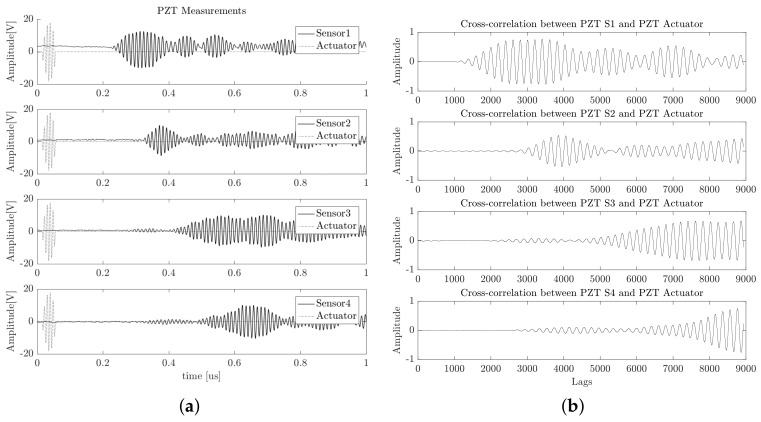
The results of the preprocessing stage of the guided wave structural response. (**a**) Raw data. (**b**) Cross-correlated signals.

Several applications for structural damage assessment have demonstrated the effectiveness of using cross-correlation signals [22]. For example, in [23], the authors use damage identification methods based on the natural excitation technique (NeXT), which employ cross-correlation signals for modal analysis, which has been useful for damage identification in civil structures. Likewise, the advantage of processing data correlation in the discrete frequency domain with methods such the eigen realization algorithm (ERA) is exploited for damage condition assessments in civil structures, where changes in stiffness and damping properties are identified regardless of the influence of hysteretic and non-linear responses [24]. Another proposal [25] includes the estimation of the time of flight of wave packages by means of cross correlation signals to locate defects within a large area of a thin-plate specimen. The cross-correlation function between two signals Y(t) and Z(t) is defined as in Equation (1).

(1) $$R_{YZ}\left(t,t+\tau \right)=\lim_{N\to \infty }1/N\sum_{k=1}^{N}Y_{k}\left(t\right)Z_{k}\left(t+\tau \right)$$

where *N* is the number of signal samples, and τ is the lag time interval used to compute the cross-correlation signal. In our case of the PCA-based piezodiagnostics approach for damage detection, the cross-correlation is computed between the actuating signal Y(t) and the respective PZT sensor measurement Z(t). An example of cross-correlation signals corresponding to four different PZTs are illustrated in Figure 7.

The signals presented in Figure 7 belong to PZT measurements of increasing positions (i.e., PZT${}_{4}$ is further than PZT${}_{3}$, and PZT${}_{3}$ is further than PZT${}_{2}$, and so on). The profile of the computed cross-correlated signals indicates that information about the time of flight is preserved from raw time measurements. If the smoothed tone-burst signal generated by the piezoelectric actuator device is stated in the form of Equation (2), it can be deduced that the implicit arrival time is present in the PZT sensor response and can be mathematically represented by Equation (3) [25].

(2) $$S_{T}\left(t\right)=S_{0}\left(t\right)\cos\left(2\pi f_{c}t\right)\left(2\right)$$

where $S_{0}\left(t\right)$ is a short-duration smoothing window applied to the carrier signal of frequency $f_{c}$ between 0 and $t_{p}$. The total signal received at point *P* by a PZT sensor can be expressed by Equation (3).

(3) $$S_{P}\left(t\right)=\sum_{m=0}^{M-1}A_{r,m}S_{T}\left(t-t_{d,m}\right)$$

where $A_{r,m}$ represents the decreasing of the wave amplitude due to the omni-directional 2-D radiation, and $t_{d,m}$ is the arrival time delay due to the travel distance between the reference PZT (m = 0, i.e., actuator) and the point *P* with no dissipation (i.e., the wave energy conservation is assumed). It is assumed that a distance d between two consecutive PZT’s of the array, which is much smaller than the distance *r* to a generic far-distance point, P(d<<r). Additional advantages of using cross-correlation signals relies on frequency interpretation, which can also be analyzed as a convolution filter. In terms of cross power spectral density, the cross-correlation function between two time discrete signals Y(n) and Z(n) is defined as in Equation (4), where N is the number of points in the cross correlation function

(4) $$R_{YZ}\left(n\right)=\frac{1}{N}\sum_{k=1}^{N}S_{YZ}\left(k\right)e^{\frac{j2\pi nk}{N}}.$$

According to Equation (4), the cross-correlation function is an average sum of N cross-spectral densities $S_{YZ}\left(k\right)$, which filters out high frequency disturbances caused by outliers. Thus, a smoothed version of the dynamical structure response is obtained, with cleansed data and outliers removed or minimized.

#### 2.2.2. Principal Component Analysis: Baseline Model Building

**Figure 8:**
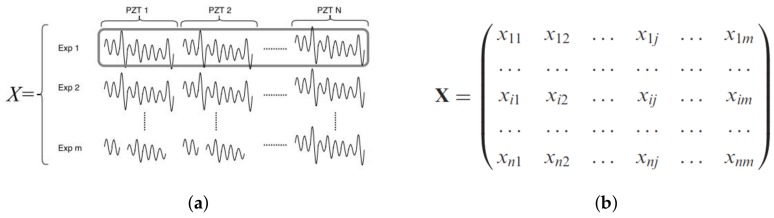
Undamaged cross-correlated baseline matrix. (**a**) Snapshot representation. (**b**) Matrix notation.

The objective of PCA is to reduce the dimensionality of a data set by preserving the data variation as much as possible. In this sense, a large number of interrelated variables can be represented in a new reduced space of coordinates with minimal redundancy. This reduced representation achieves a baseline model respect to a reference state, which has been referred to as the undamaged stage [26]. In this work, PCA is used to represent the cross-correlated signals in the reduced space, regarding the dynamical response of the structure in the undamaged state, which allows for a comparison to unknown states (possible damages). The application of PCA is performed through the following five levels:

1. Level 1—Data Organization: In this part, cross-correlated signals of each PZT sensor belonging to several repetitions of the undamaged structural state are organized in an unfolded data matrix (X) (Figure 8).
  The collected data are arranged in an n×m·K matrix, which contains information from *m* sensors and *n* experimental trials. *K* is the number of time samples recorded in the *i*-th experiment repetition. Thus, each row vector $\left(x_{i}\right)$ represents measurements from all sensors (experiment trial), and each column vector $\left(v_{j}\right)$ represents measurements from one sensor in the whole set of experiment trials at a specific time instant.
2. Level 2—Data Normalization: The undamaged cross-correlated baseline matrix is normalized in order to avoid scaling and bias issues and to reduce the influence of different sources of variability. In this work, normalization is computed by means of group scaling (GS), where each data point from the undamaged cross-correlated baseline matrix (*X*) is scaled by considering changes between sensors and the nature of the data by estimating the standard deviation for each block of piezo measurements [27]. Thus, a normalized data matrix $\bar{X}$ is obtained by standardizing *X* using the mean of each time sample for every experiment and the standard deviation of each sensor sample vector, where each ${\bar{x}}_{ijk}$ element is determined by Equation (5).
  (5) $${\hat{\mu }}_{jk}=\frac{\sum_{i}^{n}x_{ijk}}{n};\sigma_{j}=\frac{\sum_{i}^{n}\sum_{k}^{K}x_{ijk}}{n\cdot K};{\hat{x}}_{ijk}=\frac{x_{ijk}-{\hat{\mu }}_{jk}}{{\hat{\sigma }}_{j}}$$
  where $\sigma_{j}$ is the standard deviation per PZT sensor and $\mu_{ij}$ is the mean value at a specific time instant of undamaged cross-correlated baseline matrix *X*.
3. Level 3—Optimal Basis Representation: The next step is to find a set of *r* basis vectors (*P*) that satisfies the extreme value problem established by Equation (6), in order to minimize the fitness function $\varepsilon^{2}$ [28].
  (6) $$\min_{P_{i}}\varepsilon^{2}\left(r\right)=E\{||\hat{X}-\hat{X}{\left(r\right)||}^{2}\}s.t.P_{i}^{T}P_{j}=\delta_{i,j}i,j=1,2,\ldots ,r.$$
  The basis vector *P* can be estimated by computing the singular value decomposition of the covariance matrix $C_{x}$ established by Equation (7), which can be solved by using NIPALS, POD, or QR procedures [29].
  (7) $$C_{x}P=P\lambda ,\;\mathrm{where}\;C_{\bar{X}}=\frac{1}{M-1}{\bar{X}}^{T}\bar{X}$$
  where *M* is the number of trial records used to estimate the covariance matrix, and λ the respective eigenvalues.
4. Level 4—Baseline modeling: As a result, a baseline model is obtained according to the PCA procedure in Equation (8). The baseline model is a reduced representation of cross-correlated piezoelectric signals of the pristine structure, arranged in the undamaged cross-correlated baseline matrix (*X*), after the normalization procedure ($\bar{X}$).
  (8) $$\bar{X}=TP^{T}+E=model+noise$$
  where the basis vectors *P* form the linear transformation matrix that relates the data matrix $\bar{X}$ to the new coordinates, and they are known as the principal components. *T* is the projected matrix to the reduced space, and the noise *E*-matrix describes the residual variance neglected by the statistical model (Equation (8)). The variances of these new coordinates’ reduced space are the singular values (λ).
5. Level 5—Damage Detection Indexes: The two statistical indexes are the squared prediction error (Q-statistic) and the Hotelling $T^{2}$ statistic. The Q-statistic, defined by Equation (9), is a lack of fit measurement between the current experiment and the baseline records.
  (9) $$Q=\sum_{j}{\left(e_{j}\right)}^{2}$$
  where $e_{j}$ is the residual error for each j-th principal component used to reconstruct the trial experiment. The Hoteling $T^{2}$ statistic, defined by Equation (10), indicates how far each trial is from the center (T = 0) of the reduced space of the coordinates.
  (10) $$T^{2}=\sum_{j=1}^{r}\frac{t_{sij}^{2}}{\lambda_{j}}=T^{{}^{\prime }}\lambda^{-1}T.$$
  The diagnostics can thus be achieved using $T^{2}$*vs. Q* indexes. The scatter plot is an easy way of representing the information obtained from these indexes; however, some types of damages and possible boundaries can be masked. For this reason, a clustering learning algorithm is used to evaluate the influence of the preprocessing stage in the damage detection procedure.

### 2.3. Clustering Analysis: Evaluation of Condition Monitoring Quality

**Figure 9:**
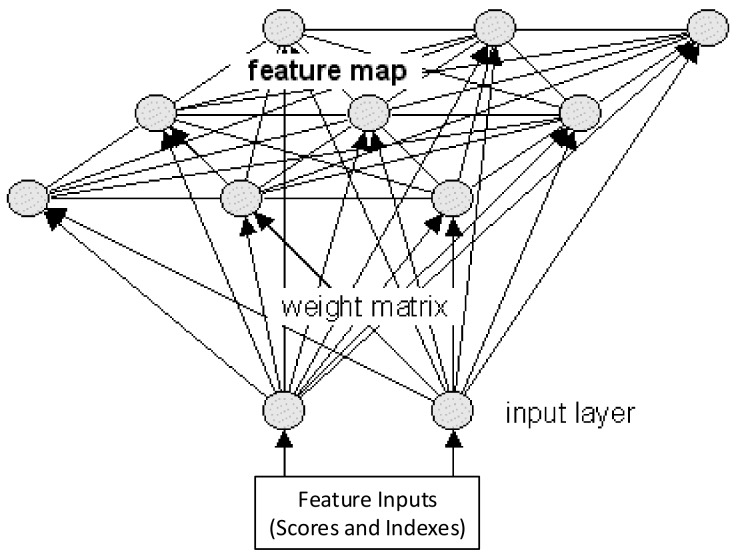
SOM clustering.

The last step is performed using one of the most commonly used unsupervised learning algorithm: a self-organizing map (SOM). The clustering process by means of an SOM is implemented in order to evaluate the results obtained through PCA-based piezodiagnostic approach when cross-correlation preprocessing is included. The SOM network consists of *N* clusters, characterized by a prototype vector (Codebook) or cluster center, which group similar cases. This clustering is achieved by means of competitive learning and preserving topology. Accordingly, nearby data in the input space are mapped into neighbor clusters [30]. Figure 9 deploys the operation of an SOM network, where the input space or feature inputs is specified by $T^{2}$ and *Q*-indexes.

The SOM quality is evaluated with quantization, topographic, and distortion error measures. The quantization error is the average distance between each experiment and its best matching unit (BMU). The topographic error corresponds to the proportion between data vectors whose first and second BMUs are not adjacent clusters and the total number of experiments. Finally, the distortion measure can be interpreted as the energy function that is minimized by the SOM. In addition, the BMU clusters are used to measure the similarity of damage types by means of validation error obtained through majority voting. In this sense, similar cases are labeled in clusters, where each label keeps only one instance and the number of stored cases. Similarly, the validation cases are ticked assigning the label with the most instances and with the most similar clusters to find the BMUs. In consequence, the validation error can be estimated by majority voting. Thus, for training purposes, 70% of the data are used for SOM parameter tuning and the remaining 30% are used to estimate the validation error.

## 3. Damage Assessment Methodology

**Figure 10:**
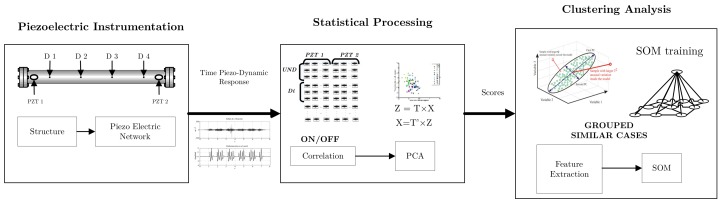
Damage assessment methodology.

Foundations of damage detection methodology used in this work are detailed in the previous section. The non-intrusive structural damage assessment methodology based on the previous constitutive elements is depicted in Figure 10. This methodology is composed of three main steps: 1. sensor signals recorded by piezoelectric instrumentation in the three experimental setups; 2. statistical processing; and 3. clustering analysis.

Two main goals are developed by implementing the methodology in Figure 10: damage detection and damage type clustering. The first task is achieved by means of statistical processing, while the second is accomplished by unsupervised learning tools. The next section details the procedure to manage these two previous goals.

### 3.1. Damage Detection Procedure

**Figure 11:**
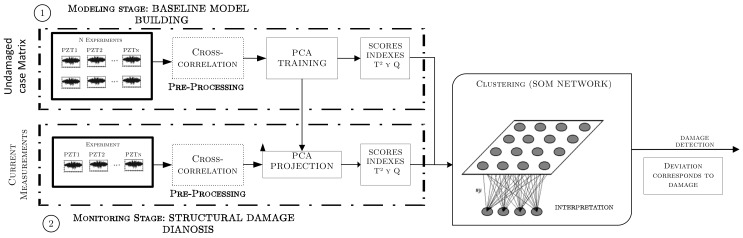
The damage detection approach.

The integration of the first two tools (statistical processing and PCA) allows for damage detection (depicted in Figure 11), where clearly the modeling and monitoring procedure can be identified. The modeling phase builds the baseline model by applying PCA to the undamaged cross-correlated baseline matrix, while the monitoring phase refers to the projection of current signals to the baseline model. Since current measurements stands for unknown structural states, two statistical indexes are computed to distinguish possible abnormal conditions, where abrupt changes of them can be associated to a structural damage.

### 3.2. General Scheme of Damage Assessment

Damage detection is achieved in the two first steps: sensor signals recorded by piezoelectric instrumentation in three experimental setups and statistical processing based on PCA. The first step consists in guided wave generation using PZT devices in order to distinguish between damaged and undamaged states. Some applications of piezodiagnostics include the detection of damage in aircraft joints [31], the detection of damage in composite panels [32], the detection of cracks and corrosion in macro-fiber composites [33], and pipeline leak assessment [34]. Likewise, the second step of PCA can be summarized as a mathematical tool widely used for feature extraction and pattern recognition [35], with several proposed methodologies for structural damage detection, such as the detection of damage and its location in structures such as pipes, wind turbines, and aircraft sections [36,37,38]. In particular, this work acquires several repetitions of PZT measurements when the structure operates in healthy conditions, and the initial trends of PZT measurements are then removed in order to compute the cross-correlation between actuating signal and measurements from PZT sensors. Thereafter, the cross-correlated data is organized in an unfolded matrix and normalized. Afterward, the singular value decomposition is computed by PCA to build the baseline model. The first *r* principal components are selected in order to obtain a reduced representation of the undamaged structural condition. In this way, the methodology builds a baseline representation of the undamaged condition of the structure, and the current condition is projected in order to determine the stage of the structure. Scatter plots of damage indexes are used to distinguish between undamaged and damaged conditions. Finally, a clustering analysis stage is achieved by using an SOM network, which demonstrates the benefits of using cross-correlation as a preprocessing stage, evaluated through the performance of clustering indexes.

## 4. Results and Discussion

To determine the consistency and effectiveness of structural damage assessment methodology, data cleansing and filtering, and structural damage detection were performed.

First, spectra was analyzed to evaluate the data cleansing and filtering properties of the cross correlation analysis. A preliminary test and was performed to detect data anomalies. Afterward, different damage scenarios were evaluated according to the methodology explained in Section 3. The main goal of this paper is to demonstrate cross-correlation functions at the preprocessing stage, for a better boundary between damage cases. In the following sections, these experimental results are presented and discussed.

### 4.1. Data Cleansing and Filtering

This item describes results intended to demonstrate the applicability of the preprocessing stage based on cross-correlation in order to minimize the adverse influence of noisy data. For this purpose, experimental data regarding the pipe section in Figure 3 were analyzed. In this experiment, four PZTs were used to sense the guide wave produced by a PZT located at the end of the pipe section and excited by an 80 KHz burst signal every second. One hundred repetitions of the experiment were conducted and recorded for the undamaged state by using a sample time Ts = 56 ns. In this way, the potential advantages of using cross-correlation for data cleansing and filtering were explored by analyzing the measurements from the undamaged state.

#### 4.1.1. Filtering

**Figure 12:**
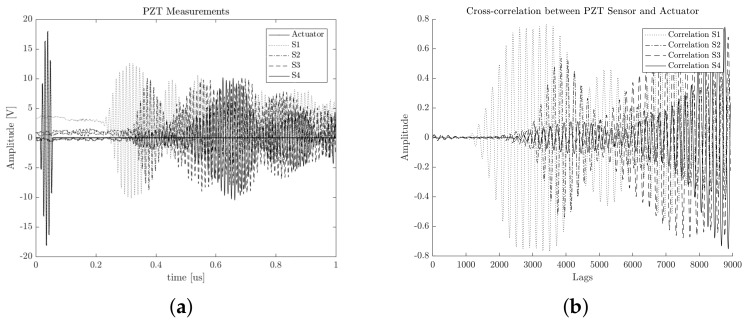
Experimental acquired signals. (**a**) Raw data from PZT sensors. (**b**) Cross-correlation signals.

Spectra was analyzed in order to verify that information in the frequency domain was preserved. Figure 12 presents an example of the recorded signals for each PZT and their respective cross-correlated signals with respect to the actuation signal.

**Figure 13:**
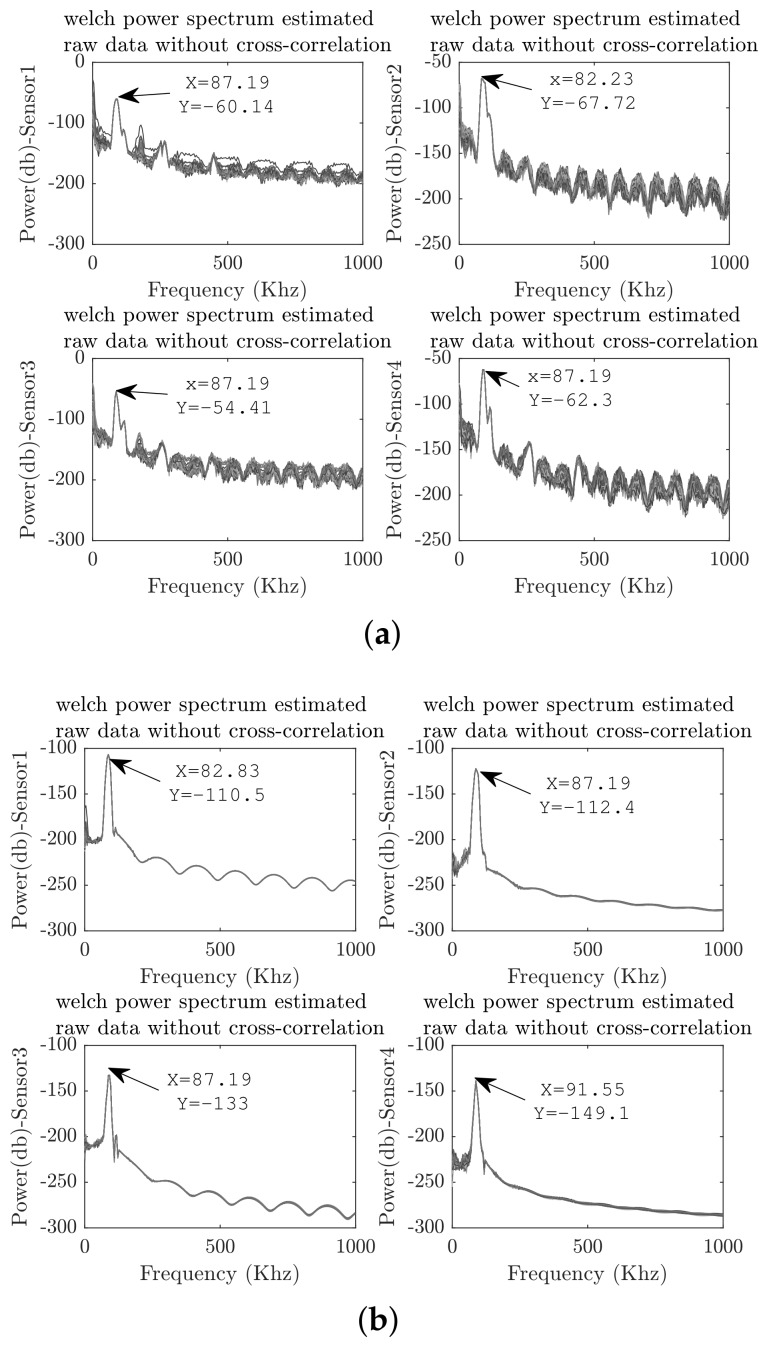
Power spectrum. (**a**) PSD from raw data PZT measurements. (**b**) Cross-PSD from correlation signals.

According to Figure 12, it can be observed that cross-correlation reduced the offset signal. The figure is a smoothed representation of dynamical behavior. The above result can be confirmed by estimating the power spectrum, which is illustrated in Figure 13 for all 100 acquired signals.

As shown in Figure 13, high frequency noise was reduced by the attenuation of the high-order harmonics. Thus, the power density of secondary side lobules in the power spectrum was reduced. As a result, the consistency of frequency information was preserved by means of an average spectrum with the same central frequency. The common offset values are excluded from signal representation. In this sense, the cross-correlation function is an effective filtering technique to be applied to piezoelectric measurements.

#### 4.1.2. Data Anomaly Detection

**Figure 14:**
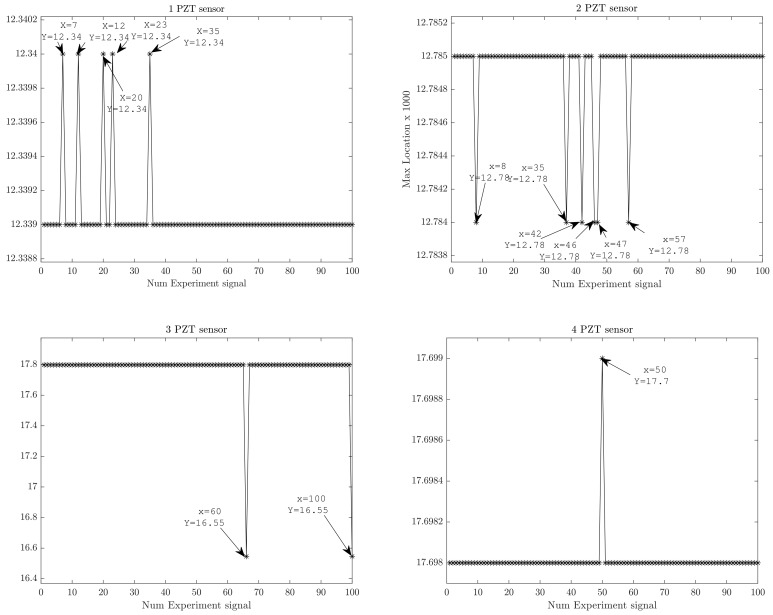
Indexes of cross-correlation maximum values.

Cross-correlation analysis is also useful as a data anomaly detection tool. For this purpose, information about the occurrence of maximum values of the cross-correlation signal can be used. Thus, the locations at which maximum cross-correlations are found were plotted in order to find possible outliers. Figure 14 shows the index location for maximum values of cross-correlation piezo measurements, where each value is associated with only one of the 100 experiments.

**Figure 15:**
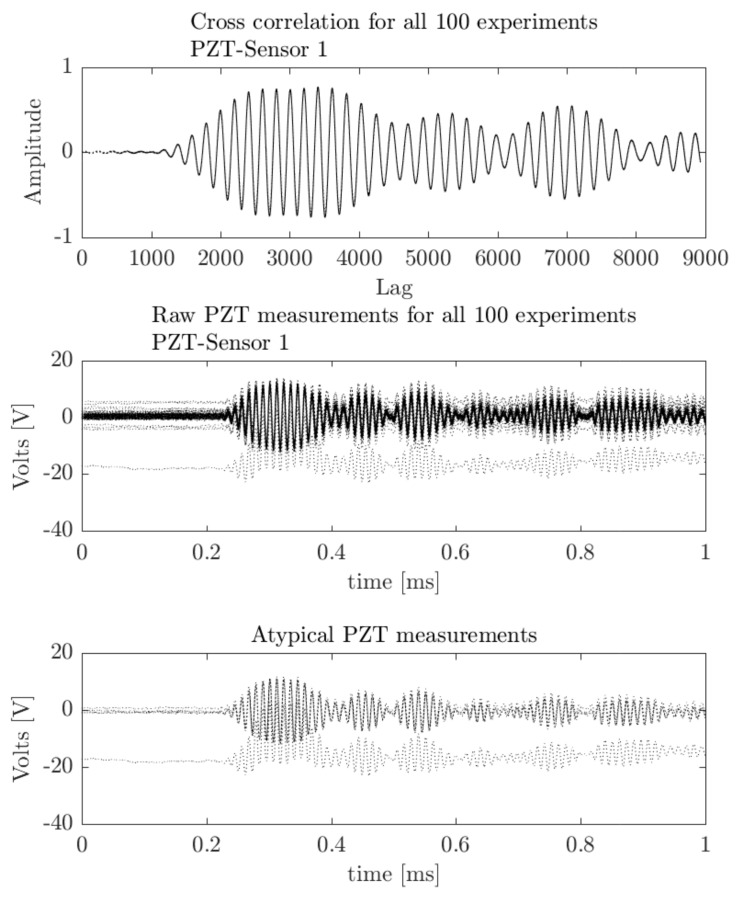
Cross-correlation signals from outliers.

**Figure 16:**
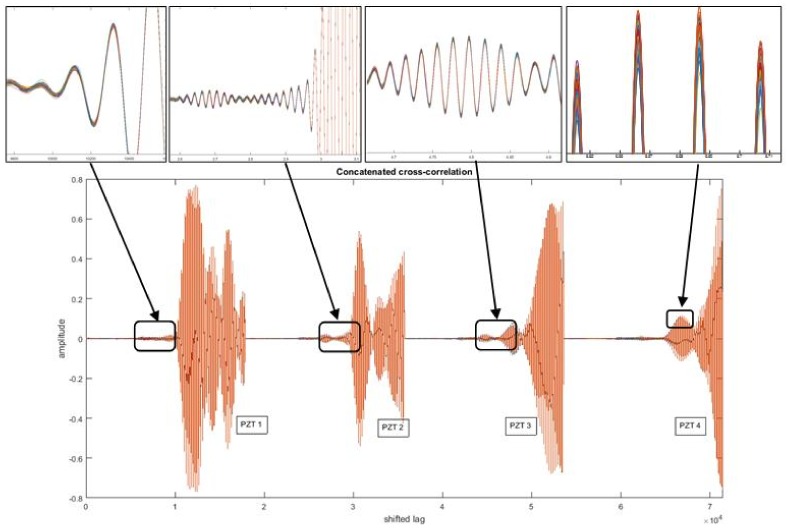
Concatenated Cross-Correlation Signals.

In Figure 14 the maximum cross-correlation values are located in the same lag position. Thus, possible abnormal data measurements can be associated with deviations of max positions. In Figure 15 possible outliers from the five measurement signals (i.e., 7, 12, 20, 23, and 35 indexes) from PZT Sensor 1 can be identified. The outliers according to information extracted from cross-correlation are depicted in Figure 16 and can be associated to offset values and trends. However, according to the upper subplot, the cross-correlation filters these atypical signals, which results in a well-defined pattern for all 100 experiment repetitions. Thus, the structural dynamical response due to guided waves is characterized by the mode conversion and low amplitude changes, as shown in Figure 16, where variations of concatenated cross-correlation signals are highlighted.

### 4.2. Structural Damage Detection

This section is intended to illustrate how the preprocessing technique based on cross-correlation signals improves the results of structural damage and diagnosis algorithms. Several experiments were conducted to show its suitability by considering different damage scenarios over the three previously described structures.

#### 4.2.1. Pipe Section Experiment

**Figure 17:**
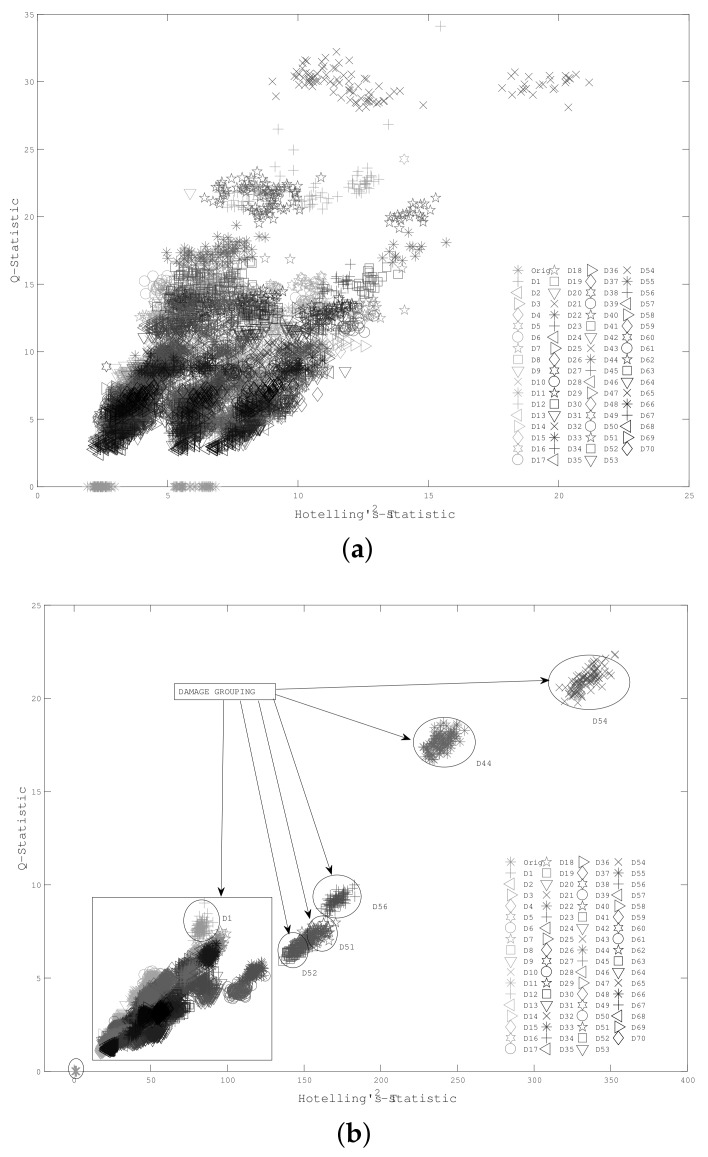
Damage indexes (**a**) without cross-correlation and (**b**) including cross-correlation.

As a first scenario, mass-added damage was considered according to experiment set up in Figure 4. Thus, two piezoelectric devices (sensor-actuator) were attached near to the bridles in the pipe section. Seventy damage classes were recreated in the test specimen by consecutive displacements of the mass along the structure. Each damage scenario, (denominated D1, D2, …, D70), belongs to a mass located at 1 cm, 2 cm, and so on, with respect to the PZT actuator. Experiments related to pristine structure cases are labeled as ‘Orig’. A number of 100 experiments per condition (damaged/undamaged) were conducted. A guided wave was induced by applying a five-cycle, 80 kHz burst type pulse on the PZT located at one end of the pipe section. The resulting $T^{2}vs.Q$ scatter plot is depicted in Figure 17, for both cases: with and without cross-correlation analysis.

According to Figure 17, by including cross correlation, some damage clusters can be distinguished in a way that they cannot be when raw PZT measurements are processed. Additionally, a clear boundary for the undamaged condition was obtained, which facilitates the damage detection process. The proposed methodology requires additional algorithms in order to manage damage localization and quantification tasks. Some approaches, such as case-based reasoning (CBR) [39], can be adapted for this. SOMs quantify and locate the damages, taking advantage of distance-based similarity measures and information retrieved from clusters obtained through damaged cases. Since the combination of the cross-correlation preprocessing stage and PCA-based piezodiagnostics results in highly distinguishable damage clusters, the use of CBR methodologies becomes feasible as a complementary tool for damage localization and quantification, which should be considered in future work.

**Figure 18:**
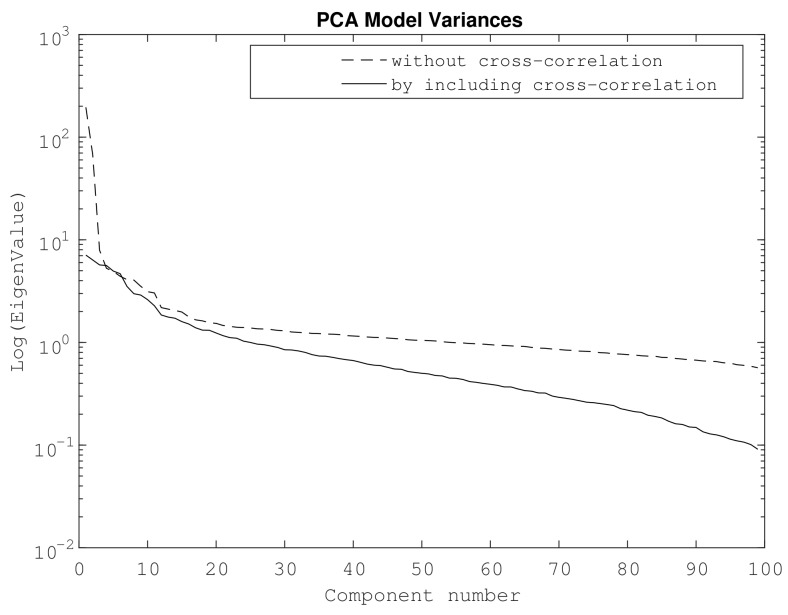
PCA model variances for mass-added scenarios in the pipe section.

In order to analyze the influence of cross-correlation in our PCA-based piezodiagnostics approach, a comparison between PCA model variances obtained with and without cross-correlation preprocessing are depicted in Figure 18.

According to the results in Figure 18, a smoothed distribution of the variance model for each principal component was obtained for the case of cross-correlation signals. Thus, unlike the results obtained from processing raw PZT measurements, there was no abrupt change with respect to the first principal component. In this sense, the variance distribution due to cross-correlation analysis entails a better clustering of damage case data. The second example is leak damage detection using the experimental configuration of Figure 3, where five PZTs were attached along the structure. The PZT at one of the ends is used as an actuator and the remaining ones as sensors. The proposed damage configuration includes different leak sizes specified in Table 1. For each type of damage, 100 experiment repetitions were conducted, where undamaged experiments are tagged with the label ‘Orig’.

**Figure 19:**
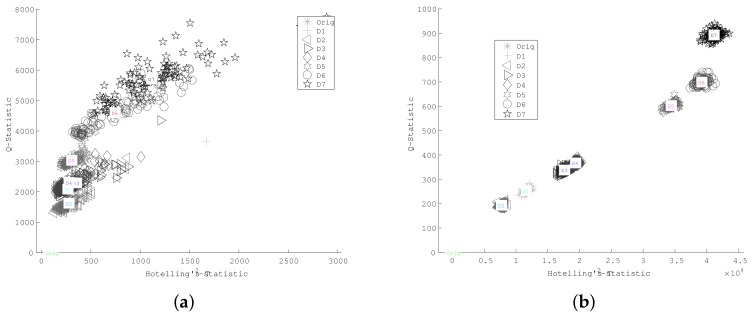
Damage indexes for leak detection (**a**) without cross-correlation and (**b**) including cross-correlation.

Figure 19 presents the resulting *Q* and $T^{2}$ statistical indexes, where a well-defined separation between different leaks combinations can be appreciated for the case of cross-correlated signals.

According to results in Figure 19, it is possible differentiate abrupt changes between the PCA index amplitudes of leaks produced through a single hole (D1, D2, D3, and D4) and those corresponding to leaks with multiple holes (D5, D6, and D7). Thus, the higher the damage index, the more severe the damage is, which allows for a simpler quantitative estimation of damage intensity by means of the PCA index magnitude. However, damage location requires additional procedures, which are being, and will continue to be, studied. Some promising approaches include the use of PCA damage indexes to estimate possible damage paths [40] and imaging methods based on the time of flight and the properties of guided waves [41,42].

**Figure 20:**
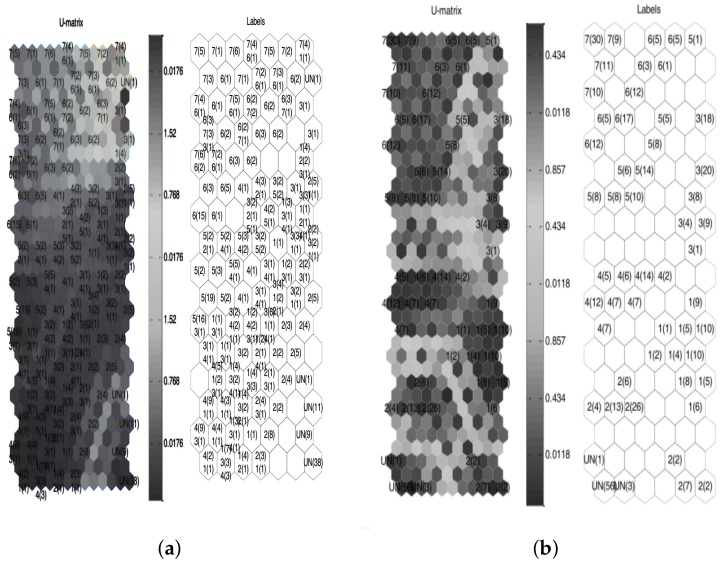
Damage indexes for leak detection (**a**) without cross-correlation and (**b**) including cross-correlation.

In order to emphasize the advantage of using cross-correlated signals, an SOM was trained by using T-squared and Q-statistics indexes as feature inputs (the same data from Figure 19), whose clusters are depicted in Figure 20.

According to Figure 20, boundaries clearly defined by empty clusters and BMU distance matrix (U-matrix) can be observed when the cross-correlation as a preprocessing stage is applied. Thus, a major differentiation between different damage types is shown. Case distribution avoids damage combinations in similar clusters, which allows for improved damage discrimination.

#### 4.2.2. Skin Panel Structure

**Figure 21:**
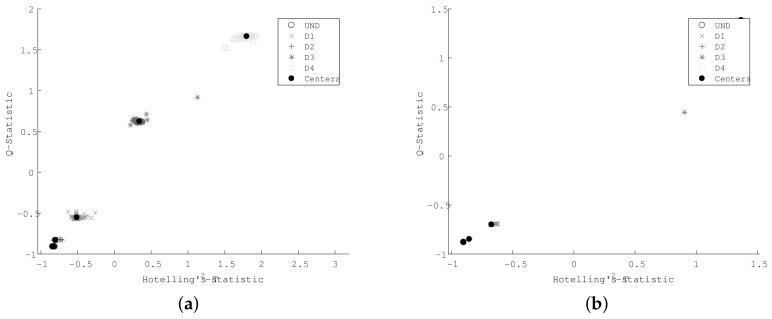
Statistical indexes for skin panel experiment (**a**) without cross-correlation and (**b**) including cross-correlation.

Experimental results for the skin panel test structure are depicted in Figure 21 using statistical indexes values and cluster centers for each damage scenario. It can be observed that major dispersion appears without correlation analysis more so than it does with it. Additionally, correlation analysis shows its efficacy in filtering atypical data-cases.

**Figure 22:**
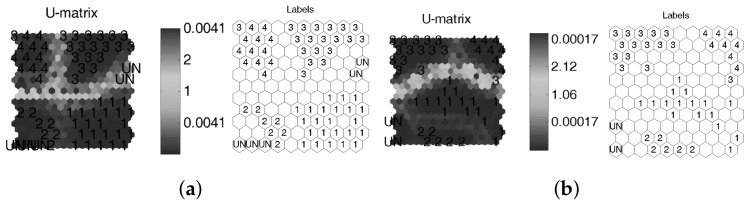
The SOM network for skin panel structure damages (**a**) without cross-correlation and (**b**) including cross-correlation.

The respective SOM network is depicted in Figure 22.

According to Figure 22, undamaged cases are separated in a better way when cross-correlation signals are used to obtain the SOM network. Additionally, the U-matrix shows a major distance values between damage cases. Table 2 summarizes the SOM quality indexes for the skin panel structure data.

#### 4.2.3. Turbine Blade Structure

**Figure 23:**
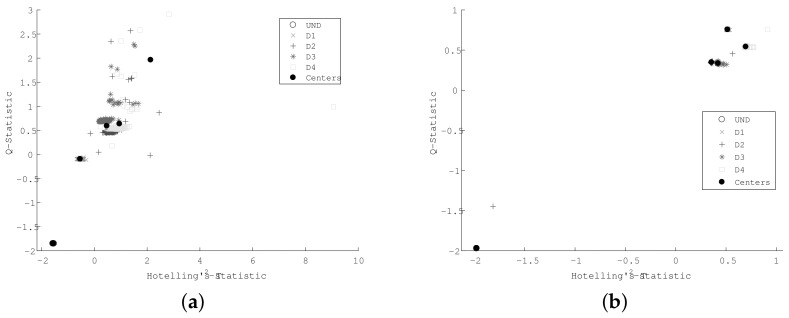
Results for the turbine blade experiment (**a**) without cross-correlation and (**b**) including cross-correlation.

Experimental results of the turbine blade test structure are depicted in Figure 23 using similar parameters of the above experiment. A clear separation between different types of damage can be highlighted when cross-correlation analysis is included, and superior performance by including cross-correlation analysis is confirmed.

**Figure 24:**
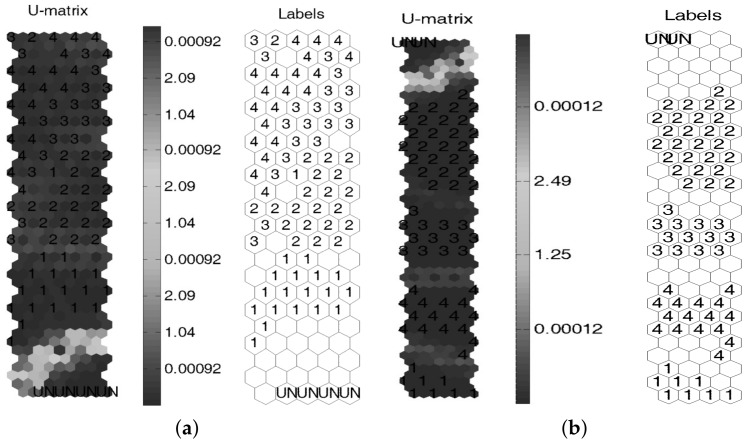
The SOM network for the turbine blade structure damages (**a**) without cross-correlation and (**b**) including cross-correlation.

The respective SOM network is presented in Figure 24.

Additionally, a better cluster separation is observed for the case when cross-correlation is used as feature inputs to the SOM network. This is validated by the SOM quality indexes summarized in Table 3, where the best indexes are obtained for the case of cross-correlated signals.

## 5. Concluding Remarks

In this paper, a robust damage assessment methodology by combining piezodiagnostics, cross-correlation signals, and PCA with capabilities of detecting structural damages was experimentally validated. The main contribution of this work is the inclusion of cross correlation as a preprocessing stage, which has become part of an integrated methodology for robust structural damage diagnosis implemented through a PCA-based piezodiagnostics approach. In this way, cross-correlation analysis is used to minimize the influence of outliers and to increase discrimination capabilities by improving the ratio of within-cluster and between-cluster distances associated to the respective damage groups. Hence, a preprocessing stage based on cross-correlated piezoelectric signals allows for adequate rejection of abnormal data. Thus, the common external noise signals are excluded in order to avoid abnormal data as well as filtering atypical cases. Additionally, better damage differentiation was obtained when a cross-correlation technique was used as a preprocessing technique. Since cross-correlation improved the clustering and differentiation of statistical indexes between damages, it was possible to distinguish damages by a simple graphical analysis. The effectiveness of the methodology was validated by analyzing experimental data from three laboratory structures, where improvements were obtained for all experiments by studying different damage types and complexity in the damage scenarios. As a main conclusion, it was demonstrated that damage diagnosis using a PCA-based piezodiagnostics scheme is highly dependent on the preprocessing stage. However, by using a correlation of piezoelectric signals, improved behavior can be obtained, with promising results for analysis of different damage types. Thus, i an integrated approach including cross-correlation analysis can be used in real world structural damage assessment tasks addressed with PCA-based piezodiagnostics. Future works should include complementary tools to manage damage localization and quantification tasks. Recommended approaches described in the state of the art include case-based reasoning and the contributions of PCA indexes, which are easily adapted and integrated to the methodology presented in this paper. Special issues regarding the optimal localization of piezoelectric sensors and the use of sparse arrays of sensors could also be studied.

## Figures and Tables

**Table 1:** Leak damage specification.

Label	Leaks (Bold = Open)	Label	Leaks (Bold = Open)
D1	**H1**, H2, H3, H4	D5	H1, H2, **H3, H4**
D2	H1, **H2**, H3, H4	D6	H1, **H2, H3, H2**
D3	H1, H2, **H3**, H4	D7	**H1, H2, H3, H4**
D4	H1, H2, H3, **H4**	*H	denotes hole

**Table 2:** SOM quality indexes for skin panel structure data.

Index	Uncorrelated Signals	Cross-Correlated Signals
Quantization error	0.0186	0.0025
Topographical error	0.0686	0.2381
Distortion measure	0.7840	0.2734
Training Error	0.5714	0
Empty Clusters	42	63
Validation Error	2.6667	1.3333

**Table 3:** SOM quality indexes for the turbine blade structure data.

Index	Uncorrelated Signals	Cross-Correlated Signals
Quantization error	0.0238	0.0021
Topographical error	0.3320	0.0362
Distortion measure	0.7840	0.1053
Training Error	6.2857	0
Empty Clusters	27	46
Validation Error	15.5556	1.3333

## Abbreviations

The following abbreviations are used in this manuscript:

AWG	Arbitrary Wave Generation
Di	Damage index i
GS	Group Scaling
NIPALS	Non-linear Iterative Partial Least Squares
NeXT	Natural Excitation Technique
PCA	Principal Component Analysis
POD	Proper Orthogonal Decomposition
PZT	Piezoelectric Device
QR	QR-Matrix Factorization
SHM	Structural Health Monitoring
UPM	Universidad Politecnica de Madrid
CBR	Case-Based Reasoning
ERA	Eigen Realization Algorithm
SOM	Self-Organizing Map
SVD	Singular Value Decomposition
